# Cognitive performance in patients with Mild Cognitive Impairment and Alzheimer's disease with white matter hyperintensities: An exploratory analysis

**DOI:** 10.1590/1980-57642016dn11-040013

**Published:** 2017

**Authors:** Maila Rossato Holz, Renata Kochhann, Patrícia Ferreira, Marina Tarrasconi, Márcia Lorena Fagundes Chaves, Rochele Paz Fonseca

**Affiliations:** 1 Mestranda do Programa de Pós-Graduação em Psicologia – Pontifícia Universidade Católica do Rio Grande do Sul (PUCRS), RS, Brazil.; 2 Aluna de Pós-doutorado do Programa de Pós-Graduação em Psicologia – PUCRS, RS, Brazil.; 3 Aluna de Graduação em Psicologia – Universidade do Vale do Rio dos Sinos, RS, Brazil.; 4 Aluna de graduação em Psicologia – PUCRS.; 5 Professora Titular do Departamento de Medicina Interna – Faculdade de Medicina da Universidade Federal do Rio Grande do Sul, RS, Brazil.; 6 Professora Adjunta do Programa de Pós-Graduação em Psicologia – PUCRS, RS, Brazil.

**Keywords:** Alzheimer's disease, Mild Cognitive Impairment, white matter hyperintensities, cognition, doença de Alzheimer, Comprometimento Cognitivo Leve, hiperintensidades de substância branca, cognição

## Abstract

**Background:**

White matter hyperintensities (WMH) are commonly associated with vascular dementia and poor executive functioning. Notwithstanding, recent findings have associated WMH with Alzheimer's disease as well as other cognitive functions, but there is no consensus.

**Objective:**

This study aimed to verify the relationship between WMH and cognitive performance in Mild Cognitive Impairment (MCI) and Alzheimer's disease (AD) patients. The study also sought to identify cognitive and demographic/cultural factors that might explain variability of WMH.

**Methods:**

The sample was composed of 40 participants (18 MCI and 22 AD patients) aged ≥ 65 years. Spearman's correlation was performed among cognitive performance (memory, language, visuospatial ability, and executive function) and WMH evaluated by the Fazekas and ARWMC scales. Two stepwise linear regressions were carried out, one with cognitive and the other with demographic/cultural variables as predictors.

**Results:**

Only naming showed significant correlation with ARWMC. Fazekas score exhibited significant correlation with all cognitive domains evaluated. Fazekas score was better predicted by episodic visual memory and age.

**Conclusion:**

This study found that the most relevant cognitive profile in MCI and AD patients with WMH was related to episodic memory. And, without taking clinical aspects into consideration, age was the best predictor of WMH.

## RESUMO.

**Introdução:**

Hiperintensidades de substância branca (HSB) são comumente associadas à demência vascular e ao mau funcionamento executivo. Contudo, descobertas recentes relacionam-no com a doença de Alzheimer, bem como com outros funcionamentos cognitivos, mas não há um consenso.

**Objetivo:**

Este estudo teve como objetivo verificar a relação entre HSB e desempenho cognitivo em pacientes com Comprometimento Cognitivo Leve (CCL) e doença de Alzheimer (DA). Assim como, identificar fatores cognitivos e demográficos / culturais que poderiam explicar a variabilidade da HSB.

**Métodos:**

A amostra foi composta por 40 participantes (18 CCL e 22 pacientes com DA) com ≥ 65 anos de idade. A correlação de Spearman foi realizada entre o desempenho cognitivo (memória, linguagem, capacidade visoespacial e função executiva) e HSB avaliada através das escalas Fazekas e ARWMC. Dois modelos de regressão linear stepwise foram realizados, um com variáveis cognitivas e outro com variáveis demográficas / culturais como preditores.

**Resultados:**

Somente nomeação apresentou correlação significativa com ARWMC. Fazekas apresentou correlação significativa com todos os domínios cognitivos avaliados. Fazekas foi melhor predito pela memória episódica visual e idade.

**Conclusão:**

Este estudo foi capaz de identificar que em pacientes com CCL e DA com HSB o perfil cognitivo mais relevante está relacionado à memória episódica. E, sem levar em consideração os aspectos clínicos, a idade foi o melhor preditor da HSB.

## INTRODUCTION

Alzheimer's disease (AD) is traditionally characterized by amyloid-beta extracellular amyloid-positive neuritic plaques and intracellular tau-positive neurofibrillary tangles. However, recent findings identify that white matter hyperintensities (WMH) could play an important role, proving even more strongly associated with preclinical AD than other AD biomarkers.^1^ WMH are commonly associated with vascular neurodegenerative diseases, which are a consequence of vascular risk factors and lead to vascular cognitive impairment.^2^ Cerebrovascular lesions are found in the majority of late-onset AD, with age representing an important factor related to cognitive impairment.^3^ Cerebrovascular lesions seem to be related to both elderly individuals with dementia and healthy subjects.^4^^,^ 5 Furthermore, the value of WMH findings in Alzheimer's disease (AD) is still controversial,^6^ because they can be found in AD patients and in vascular dementia patients. However, a recent study suggested that WMH are able to independently predict cognitive impairment in AD patients.^7^ WMH are mainly identified in frontal and parietal regions.^4^^,^ 8 and periventricular areas.^5^ The presence of WMC in the hippocampus can be supportive for memory decline, and atrophy of this region is now recognized as a good biomarker of the Alzheimer pathology.^8^^,^ 9

WMH can be classified according to severity, extent and site of injury/change.^4^^,^ 10 ^,^ 11 Brain areas with greatest and most incipient lesions are detected by neuroimaging.^4^ The most used scales for the evaluation and classification of these vascular components are the Fazekas, used to describe the severity of periventricular and deep white-matter hyperintensities;^6^ and the Age Related White Matter Changes (ARWMC) which assesses white matter lesions and basal ganglia lesions.^4^^,^ 11 Some authors suggested that WMH are associated with sociodemographic factors such as age and previous medical history of hypertension, diabetes and cerebrovascular diseases that are widely associated as risk factors for cognitive impairment.^4^^,^ 5 ^,^ 11 ^-^ 13 Although WMH have been shown to be associated with age and vascular risk factors, the exact mechanisms explaining these association remain unclear.^11^

Vascular cognitive impairment is a term used for the range of changes and impairments within a cognitive continuum associated with vascular pathologies,^10^^,^ 14 ^-^ 17 but that do not yet meet criteria for dementia. In contrast, Vascular Mild Cognitive Impairment (VaMCI) is a concept involving Mild Cognitive Impairment (MCI) that includes a clinical history of cerebrovascular disease, or diffuse WMH;^18^^-^ 20 as well as impairment in at least one cognitive domain, but without underlying functional impairment.^10^^,^ 18 ^,^ 21 Both VaMCI and MCI may have multiple cognitive components covered by the pathology, yet result in the same memory difficulties.^20^ However, dementia and MCI due to AD present initial significant episodic memory impairment which progresses to impairments in other cognitive components.^22^ The first impairments in VaMCI are related to executive functioning, semantic memory, visuospatial skills, attention and perception.^22^ Nonetheless, the executive components of processing speed, switching, and inhibitory control could be more closely associated with the extension of the WMH.^19^ Many of the studies on WMH associated with cognitive functions investigated only one specific cognitive domain, or carried out comparison with vascular dementia.^18^^,^ 21 ^,^ 22

Thus, it is known that sociocultural factors are strongly related with dementia and inherent cognitive impairment, with aging being the main risk factor for the development of dementia.^23^ Brain atrophy also increases significantly with advancing age.^12^ Thus, it is necessary to understand which structural, cognitive and sociocultural factors are related to the profile of MCI patients with greater vascular components. Therefore, there is still a lack of information allowing the establishment of this explanatory cognitive profile for patients with MCI and dementia due to AD with greater WMH. Thus, the aim of this study was to determine whether there is a relationship between the amount of vascular components (as expressed by degrees of WMH) and cognitive performance (episodic and visual memory, language, visuospatial ability and executive function) in amnestic MCI patients and patients with dementia due to AD. In an exploratory study, we estimated the strength of the association of different cognitive domains with WMH scores. We also sought to identify cognitive and demographic/cultural factors (sex, age, education, frequency of reading and writing habits) that might explain variability of vascular components.

## METHODS

### Participants.

The initial sample consisted of 55 participants, 30 MCI and 25 mild AD patients. The patients were recruited from the Dementia Clinic of the Hospital das Clínicas de Porto Alegre (HCPA) and through media advertising (television, radio and internet). The diagnostic criteria used for amnestic MCI patients were those of Winblad et al. (2004).^24^ For MCI diagnosis, patients had to have a performance of 1.5 SD below normative data on the Rey Auditory-Verbal Learning Test (delayed recall). Diagnosis of dementia due to AD was based on the current diagnostic criteria for probable or possible Alzheimer's disease of the National Institute of Neurologic and Communicative Diseases and Vascular Cerebral Accident and Alzheimer Disease Related Association (NINCDS-ADRDA) criteria.^9^^,^ 25 Only history of concomitant vascular disease was accepted for inclusion contributing to the possible clinical manifestations of AD.^26^ All other concomitant pathologies were excluded. Patients with dementia due to AD had to be classified with mild severity as assessed by the Clinical Dementia Rating Scale (CDR)^26^^,^ 27 or with a scores on the Mini-Mental State Examination (MMSE) > 14,^28^ or on the Activities of Daily Living Questionnaire (ADL-Q) < 66.^29^

Participants with sensory disturbances (auditory and/or visual) without correction; current or prior history of substance abuse or dependence; neurological disorders (epilepsy, tumor, TBI) were excluded. Mild current depressive symptoms, as assessed by the Geriatric Depression Scale (GDS-15) ≤ 6,^30^^,^ 31 were not a basis for exclusion because this symptomatology may be present in MCI patients^32^ or mask common features at the onset of dementia such as apathy.^33^

Preliminary analyses identified the influence of age as responsible for the differentiation of the amount of vascular components among MCI and dementia patients. Therefore, the analysis of this study only included patients over 65 years of age (number of excluded participants = 15). Thus, the final sample was composed of 40 elders, comprising 18 amnestic MCI and 22 patients with mild dementia due to AD.

### Procedures and instruments.

The study was approved by the Research Ethics Committee of the Pontifical Catholic University of Rio Grande do Sul (number 657.955). All participants provided written consent for participation, and a family member/caregiver also provided written consent for AD patients. All subjects were assessed individually in noise-free rooms. Assessments were carried out in up to three evaluation sessions that lasted approximately one to two hours each, under appropriate conditions in accordance with ethical guidelines for research with human participants. All interviewers were instructed to monitor patients with signs of fatigue and to interrupt the session when this appeared to be interfering with the participant's performance.

Participants answered a sociocultural and health aspects questionnaire to identify inclusion and exclusion criteria. This semi-structured questionnaire encompasses questions about sex, age, education, frequency of reading and writing habits,^34^ use of medications, description of onset and progression of forgetfulness symptoms, or functional impairment. It also assesses socioeconomic status according to the Brazilian Association of Companies and Research.^35^ The other instruments used for evaluation were divided by domain and are presented in Table 1.

**Table 1:** Instruments for cognitive evaluation.

**Global Cognition**	• Mini-Mental State Examination (MMSE): evaluates aspects of global cognition such as orientation, calculation, attention, memory and language.^36^^,^ 37 ^,^ 38
**Memory**	• Rey Auditory-Verbal Learning Test (RAVLT): we use this instrument to measure long-term episodic verbal memory (RAVLT-A7).^39^^,^ 40
	• Brief Neuropsychological Assessment Battery (NEUPSILIN): subtests of prospective memory and semantic memory.^41^
	• Consortium to Establish a Registry for Alzheimer's Disease (CERAD): subtest of praxis recall to estimate episodic visual memory.^42^
**Executive Function**	• Wechsler Adult Intelligence Scale (WAIS-III): digit span subtest to evaluate working memory.^43^^,^ 44
**Language**	• Montreal–Toulouse Language Assessment Battery (MTL): auditory comprehension and naming subtests.^45^^,^ 46
	• Montreal Communication Evaluation Battery (MAC): unconstrained, phonological and semantic verbal fluency subtests.^47^^,^ 48
**Visuospatial ability**	• Consortium to Establish a Registry for Alzheimer's Disease (CERAD): constructional praxis subtest.^42^

All participants underwent 1.5T MRI scans of the brain at the Radiology Department of HCPA. A qualified neurologist who was a specialist in the field of dementia evaluated all scans. For reliability, 30% of the scans were drawn for a blind reassessment and concordance was 100%.

Two different scales were used to evaluate WMH. The Fazekas scale measures the severity of periventricular and deep white-matter hyperintensities using a 0 to 3 point score.^6^ The Age Related White Matter Changes (ARWMC) scale assesses white matter lesions and basal ganglia lesions also using a 0 to 3 point score.^4^ On both scales, higher scores indicate more vascular component present in the brain.

### Data analysis.

Data were analyzed using the Statistical Package for Social Sciences (SPSS), version 23.0. Clinical, cognitive and demographic/cultural variables were compared between groups using the Mann-Whitney U test and Chi-square test. Spearman's correlation was performed among memory, executive function, language and visuospatial ability performance and Fazekas and ARWMC scores given the variables had a nonparametric distribution. We carried out two stepwise linear regressions. One with cognitive performance and Fazekas score, and the other with demographic/cultural aspects and Fazekas score. We only selected the four cognitive variables with highest correlation with Fazekas score for the regression model because of the sample size.^49^ Thus, episodic visual memory (praxis recall test), naming test, Mini-Mental State Examination and Clock Drawing Test were entered as independent variables in a stepwise linear regression model for Fazekas score. Age, education, sex and frequency of reading and writing habits were entered as independent variables in a stepwise linear regression model for Fazekas score. We did not carry out stepwise linear regression model for the ARWMC scale because only naming performance exhibited a significant correlation with the ARWMC scale. Results were considered significant at p < 0.05.

## RESULTS

No significant differences for the demographic/cultural and clinical variables were observed between MCI and AD dementia patients. The groups showed significant differences on 4 cognitive tests (Table 2).

**Table 2:** Participant characteristics.

Variables	MCI (n=18) M (SD)	AD (n=22) M (SD)	p*
Age	72.94 (5.15)	77.27 (5.38)	0.307
Years of education	11.56 (5.56)	7.27 (6.44)	0.060
Frequency of reading and writing habits	11.28 (6.30)	6.23 (5.09)	0.060
Socioeconomic status	31.28 (10.53)	28.05 (7.60)	0.526
Fazekas score	2.11 (1.18)	2.86 (1.61)	0.097
ARWMC score	1.11 (0.58)	1.50 (1.10)	0.081
Mini-Mental State Examination	25.44 (3.97)	21.23 (4.45)	0.060
Episodic verbal memory (RAVLT- A7)	4.94 (2.98)	1.14 (1.91)	**0.004**
Episodic visual memory (CERAD – praxis recall)	5.28 (3.30)	2.36 (3.24)	0.067
Visuospatial ability (CERAD – constructional praxis)	9.56 (1.62)	8.41 (2.04)	0.112
Clock drawing test	2.39 (1.57)	1.59 (1.53)	**0.033**
Digit span	10.94 (2.84)	9.00 (2.31)	0.399
Auditory comprehension	17.50 (1.42)	15.91 (2.09)	**0.032**
Naming test	28.17 (1.88)	26.23 (2.71)	**0.032**
Unconstrained verbal fluency	38.17 (16.60)	25.55 (14.82)	0.060
Phonological verbal fluency	15.89 (7.39)	10.67 (4.25)	0.079
Semantic verbal fluency	17.00 (5.03)	12.00 (4.17)	**0.040**
Semantic memory	4.56 (0.86)	4.45 (0.67)	0.678
Prospective memory	1.28 (0.38)	0.83 (0.74)	**0.002**
Sex – Female – n (%)	14 (77.8)	16 (72.7)	1.000

p≤0.05 indicates significance. RAVLT: Rey Auditory Verbal Learning Test, CERAD: Consortium to Establish a Registry for Alzheimer's Disease.

Table 3 shows the correlations among cognitive performance, Fazekas and ARWMC scores. Tables 4 and 5 present the predictors that best explained the variability in the Fazekas score. The more intense the vascular components, the lower the performance in episodic visual memory (explaining 17% of this variability). The older the person, the greater the vascular component present (explaining 18% of this variability).

**Table 3:** Spearman's correlation among memory, executive function, language and visuospatial ability performance and Fazekas and ARWMC scale scores.

Variables	Fazekas			ARWMC	
	rho	*p*		rho	*p*
Mini-Mental State Examination	–0.402	0.010		–0.105	0.518
Episodic verbal memory (RAVLT- A7)	–0.345	0.029		–0.235	0.145
Episodic visual memory (CERAD – praxis recall)	–0.445	0.004		–0.251	0.119
Visuospatial ability (CERAD - constructional praxis)	–0.286	0.073		–0.181	0.264
Clock drawing test	–0.366	0.020		–0.220	0.173
Digit span	–0.328	0.039		–0.190	0.240
Auditory comprehension	–0.346	0.029		–0.117	0.471
Naming test	–0.436	0.005		–0.352	0.026
Unconstrained verbal fluency	–0.338	0.033		–0.246	0.126
Phonological verbal fluency	–0.259	0.111		–0.165	0.315
Semantic verbal fluency	–0.320	0.047		–0.207	0.207
Semantic memory	–0.060	0.713		0.058	0.723
Prospective memory	–0.314	0.052		–0.069	0.678

p≤0.05 indicates significance. RAVLT: Rey Auditory Verbal Learning Test, CERAD: Consortium to Establish a Registry for Alzheimer's Disease.

**Table 4:** Stepwise linear regression of cognition and the Fazekas scale

Dependent variables	Predictors	B	SE	β	T	*p*	ΔR^2^	F Change	Sig F Change
Fazekas	**Model 1** Episodic visual memory	–0.174	0.061	–0.420	–2.849	0.007	0.176	8.118	0.007

p≤0.05 indicates significance.

**Table 5:** Stepwise linear regression of sociodemographic/cultural variables and the Fazekas scale

Dependent variables	Predictors	B	SE	β	T	*p*	ΔR^2^	F Change	Sig F Change
Fazekas	**Model 1** Age	0.112	0.038	0.430	2.940	0.006	0.185	8.643	0.006

p≤0.05 significant.

## DISCUSSION

The main objective of the study was to evaluate the relationship between the vascular component measured by the Fazekas and ARWMC scales and the cognitive evaluation in MCI and AD patients. Secondarily, an explanatory analysis was performed for both sociodemographic data and for cognitive data components in relation to the Fazekas scale. The results indicated a moderate, negative association between the Fazekas scale and the cognitive components of executive functions, episodic memory (verbal and visual) and language. The ARWMC only showed a moderate, negative association with naming performance.

The correlation analysis indicated that the Fazekas scale was associated with more cognitive factors than the ARWMC scale. The ARWMC scale showed only a moderate association with naming. In contrast with the Fazekas scale, besides periventricular and deep white matter lesions, the ARWMC scale also scores basal ganglia lesions. Although brain cortical areas are traditionally related to cognitive function including language,^50^ the basal ganglia are more related to language functioning than to other cognitive functions,^51^ which could explain the association with the naming test in our sample. On the other hand, Fazekas score correlated with the executive components of organization, visuospatial planning, verbal cognitive flexibility, in addition to unconstrained and semantic verbal fluency, auditory comprehension and naming, and episodic memory (verbal and visuospatial).

The regression analysis for the cognitive components showed that only visual episodic memory significantly explained 17% of the Fazekas variance (i.e., the more vascular component present the lower the performance in episodic visual memory). Episodic memory deficit is commonly observed in amnestic MCI patients who subsequently progress to a diagnosis of AD dementia.^52^ Memory impairment is frequently the primary cognitive deficit in patients with dementia due to AD.^9^By contrast, patients with vascular dementia showed more visuospatial difficulties than patients with probable AD.^22^ Previous studies have shown a diversified cognitive profile in patients with VaMCI depending on the characteristics of the sample compared. These studies reported impairment in executive functioning in VaMCI patients compared to normal controls^19^ and in attention/executive, memory and visuospatial functioning compared against individuals with small-vessel disease but without cognitive impairment,^53^ and decreased impairment in episodic memory (verbal and visual) and executive functioning relative to MCI patients.^54^

Regarding the sociodemographic components analyzed in the present study, age explained 18% of the variance found by the Fazekas scale. Some studies have suggested that age is strongly related to WMH.^12^^,^ 55 Together with age, WMH are risk factors for the development of neurodegenerative diseases such as AD.^23^^,^ 55 However, the explanatory model shows that age has a small percentage of explanation. This was expected because clinical components such as hypertension, diabetes, previous stroke, among others, probably better explain the increase in white matter damage since they are risk factors for greater damage and risk of cerebrovascular diseases.^4^^,^ 11 ^,^ 13 Additionally, studies have shown that small-vessel cerebrovascular disease potentially contributes to the pathogenesis of AD,^56^ while WMH contribute to brain atrophy patterns in regions related to Alzheimer's disease dementia^57^ and can be more strongly associated with preclinical AD than other AD biomarkers.^1^

Limitations of the present study should be considered. The small sample size and the use of the clinical diagnosis for the classification of amnestic MCI and dementia due to AD patients are probably important. We used the Fazekas and ARWMC scales to estimate number, location and intensity of WMH – not volume of WMH – which are less affected by brain volume measurements (and cerebral atrophy). However, the cortical thickness estimation for atrophy would control an important confounder for volume of WMH. Furthermore, studies with the inclusion of AD biomarkers would better classify patients into more specific clinical groups.

Surprisingly, the study was able to identify, even for patients with profiles of WMH, that the most relevant cognitive profile was related to episodic memory. This finding supports verbal and visual episodic memory as the main cognitive components to be evaluated in MCI and dementia due to AD patients. Further studies that explore specific cognitive components to detect vascular components in dementia would aid in interventions and orientations.

## References

[1] Kandel BM, Avants BB, Gee JC, McMillan CT, Erus G, Doshi J (2016). White matter hyperintensities are more highly associated with preclinical Alzheimer’s disease than imaging and cognitive markers of neurodegeneration. Alzheimers Dement (Amst).

[2] Chui HC, Ramirez-Gomez L (2015). Clinical and imaging features of mixed Alzheimer and vascular pathologies. Alzheimers Res Ther.

[3] Attems J, Jellinger KA (2014). The overlap between vascular disease and Alzheimer’s disease - lessons from pathology. BMC Med.

[4] Wahlund LO, Barkhof F, Fazekas F, Bronge L, Augustin M, Sjögren M (2001). A New Rating Scale for Age-Related White Matter Changes Applicable to MRI and CT. Stroke.

[5] Targosz-gajnia M, Siuda J, Ochudlo S, Opala G (2009). Cerebral white matter lesions in patients with dementia - from MCI to severe Alzheimer’s disease. J Neurol Sci.

[6] Fazekas F, Chawluk JB, Alavi A, Hurtig HI, Zimmerman RA (1987). MR Signal Abnormalities at 1 5 T in Alzheimer’s Dementia and Normal Aging. AJR Am J Roentgenol.

[7] Gordon BA, Najmi S, Hsu P, Roe CM, Morris JC, Benzinger TLS (2015). The effects of white matter hyperintensities and amyloid deposition on Alzheimer dementia. NeuroImage Clin.

[8] Leeuw FE, Barkhof F, Scheltens P (2004). White matter lesions and hippocampal atrophy in Alzheimer’s disease. Neurology.

[9] McKhann GM, Knopman DS, Chertkow H, Hyman BT, Jack CRJ, Kawas CH (2011). The diagnosis of dementia due to Alzheimer’s disease recommendations from the National Institute on Aging-Alzheimer’s Association workgroups on diagnostic guidelines for Alzheimer’s disease. Alzheimer Dement.

[10] Sudo FK, Alves GS, Tiel C, Ericeira-Valente L, Moreira DM, Laks J, Engelhardt E (2015). Neuroimaging criteria and cognitive performance in vascular mild cognitive impairment A systematic review. Dement Neuropsychol.

[11] Xiong YY, Mok V (2011). Age-Related White Matter Changes. J Aging Res.

[12] Brugulat-serrat A, Rojas S, Bargalló N, Conesa G, Minguillón C, Fauria K (2017). Incidental findings on brain MRI of cognitively normal first-degree descendants of patients with Alzheimer’s disease a cross-sectional analysis from the ALFA (Alzheimer and Families) project. BMJ Open.

[13] Fujishima M, Kiyohara Y (2002). Incidence and risk factors of dementia in a defined elderly japanese population the hisayama study. Ann New York Acad Sci.

[14] Garret KD, Browndyke JN, Whelihan W, Paul RH, DiCarlo M, Moser DJ (2004). The neuropsychological profile of vascular cognitive impairment - no dementia comparisons to patients at risk for cerebrovascular disease and vascular dementia. Arch Clin Neuropsychol.

[15] Brien JTO, Psych FRC, Brien O (2006). Vascular Cognitive Impairment. Am J Geriatr Psychiatry.

[16] Skrobot OA, Brien JO, Black S, Chen C, Decarli C, Erkinjuntti T (2017). The Vascular Impairment of Cognition Classification Consensus Study. Alzheimers Dement.

[17] Suvarna A (2009). Vascular cognitive impairment. Indian J Psychiatry.

[18] Gauthier S, Rockwood K (2003). Does Vascular MCI Progress at a Different Rate Than Does Amnestic MCI. Int Psychogeriatr.

[19] Sudo FK, Eduardo C, Alves O, Alves GS, Ericeira-valente L, Tiel C (2013). White matter hyperintensities , executive function and global cognitive performance in vascular mild cognitive impairment. Arq Neuropsiquiatr.

[20] Yu Y, Zhao W, Li S, Yin C (2017). MRI-based comparative study of different mild cognitive impairment subtypes: protocol for an observational case - control study. BMJ Open.

[21] Petersen RC, Caracciolo B, Brayne C, Gauthier S, Jelic V, Fratiglioni L (2014). Mild cognitive impairment A concept in evolution. J Int Med.

[22] Graham NL, Emery T, Hodges JR (2004). Distinctive cognitive profiles in Alzheimer’s disease and subcortical vascular dementia. J Neurol Neurosurg Psychiatry.

[23] Kaiser NC, Miller KJ, Siddarth P, Ercoli LM, Small GW (2013). The impact of age and Alzheimer’s disease risk factors on memory performance over time. Aging Health.

[24] Winblad B, Palmer K, Kivipelto M, Jelic V, Fratiglioni L, Wahlund LO (2004). Mild cognitive impairment - beyond controversies, towards a consensus report of the International Working Group on Mild Cognitive Impairment. J Int Med.

[25] Frota NAF, Nitrini R, Damasceno BP, Forlenza O, Dias-Tosta E, Silva AB (2011). Critérios para o diagnóstico de doença de Alzheimer. Dement Neuropsychol.

[26] Hughes CP, Berg L, Danziger WL, Coben LA, Martin RL (1958). A new clinical scale for the staging of dementia. Br J Psychiatr.

[27] Chaves ML, Godinho C, Porto CS, Mansur L, Carthery-Goulart MT, Yassuda MS, Beato R (2011). Cognitive, functional and behavioral assessment Alzheimers disease. Dement Neuropsychol.

[28] Reisberg B, Jamil IA, Khan S, Monteiro I, Torossian C, Ferris S, Abou-Saleh MT, Katona C, Kuma A (2011). Starting dementia. Principles and Practice of Geriatric Psychiatry.

[29] Medeiros ME, Guerra RO (2009). Translation, cultural adaptation and psychometric analysis of the Activities of Daily Living Questionnaire (ADLQ) for functional assessment of patients with Alzheimer’s disease. Rev Bras Fisiot.

[30] Yesavage JA, Brink TL, Rose TL, Lum O, Huang V, Adey M, Leirer VO (1982). Development and validation of a geriatric depression screening scale a preliminary report. J Psychiatric Res.

[31] Almeida OP, Almeida SA (1999). Confiabilidade da versão brasileira da escala de depressão em geriatria (GDS) versão reduzida. Arq Neuropsiquiatr.

[32] Heser K, Tebarth F, Wiese B, Eisele M, Bickel H, Ko M (2013). Age of major depression onset , depressive symptoms , and risk for subsequent dementia results of the German Study on Ageing, Cognition, and Dementia in Primary Care Patients (AgeCoDe). Psychol Med.

[33] McPherson S, Fairbanks L, Tiken S, Cummings JL, Back-Madruga C (2002). Apathy and executive function in Alzheimer’s disease. J Int Neuropsychol Soc.

[34] Pawlowski J, Remor E, Parente MAMP, de Salles JF, Fonseca RP, Bandeira DR (2002). The influence of reading and writing habits associated with education on the neuropsychological performance of Brazilian adults. Read Writ.

[35] ABEP - Associação Brasileira das Empresas de Pesquisa Critério de Classificação Econômica Brasil.

[36] Folstein MF, Folstein SE, McHugh PR (1975). "Mini-Mental State": a practical method for grading the cognitive state of patients for the clinician. J Psychiatr Res.

[37] Chaves MLF, Izquierdo IA (1992). Differential diagnosis between dementia and depression a study of efficiency increment. Acta Neurol Scand.

[38] Kochhann R, Varela JS, Lisboa CSM, Chaves MLF (2010). The Mini Mental State Examination Review of cutoff points adjusted for schooling in a large Southern Brazilian sample. Dement Neuropsychol.

[39] Rey A (1958). L’examenclinique en psychologie.

[40] Malloy-Diniz LF, Lasmar VAP, Gazinelli LSR, Fuentes D, Salgado JV (2007). The Rey auditory-verbal learning test applicability for the Brazilian elderly population. Rev Bras Psiq.

[41] Fonseca RP, Salles JF, Parente MAMP (2009). Neupsilin - Instrumento de Avaliação Neuropsicológica Breve.

[42] Bertolucci PH, Okamoto IH, Brucki SM, Siviero MO, Toniolo NJ (2001). Ramos LR Applicability of the CERAD neuropsychological battery to Brazilian elderly. Arq Neuropsiquiatr.

[43] Wechsler D (1997). WAIS-III: administration and scoring manual.

[44] Nascimento E (2004). WAIS III - Escala de inteligência Wechsler para adultos.

[45] Nespoulous JL, Lecours AR, Lafond D (1986). MT 86 - Protocole Montréal-Toulouse d’examen linguistique de l’aphasie.

[46] Parente MA, Fonseca RP, Pagliarin K, Barreto S, Siqueira E, Hubner L, Joanette Y (2016). MTL BRASIL - Bateria Montreal Toulouse de Avaliação da Linguagem.

[47] Joanette Y, Ska B, Côté H (2004). Protocole MEC - Protocole Montreál d’Évaluation de la Communication.

[48] Fonseca RP, Parente MAMP, Cote H, Ska B, Joanette Y (2008). Bateria MAC - Bateria Montreal de Avaliação da Comunicação.

[49] Field A (2009). Descobrindo a Estatística Utilizando o SPSS.

[50] Chen C, Omiya Y (2014). Brain asymmetric in cortical thickness is correlated with cognitive function. Front Hum Neurosci.

[51] Leisman G, Braun-Benjamin O, Melillo R (2014). Cognitive-motor interactions of the basal ganglia in development. Front Syst Neurosci.

[52] Albert MS, DeKosky ST, Dickson D, Dubois B, Feldman HH, Fox NC (2011). The diagnosis of mild cognitive impairment due to Alzheimer’s disease Recommendations from the National Institute on Aging-Alzheimer’s Association workgroups on diagnostic guidelines for Alzheimer’s disease. Alzheimer’s & Dementia?: J Alzheimers Assoc.

[53] Cao WW, Wang Y, Dong Q, Chen X, Li YS, Zhou Y (2017). Deep microbleeds and periventricular white matter disintegrity are independent predictors of attention/executive dysfunction in non-dementia patients with small vessel disease. Int Psychogeriatr.

[54] Divya KP, Menon RN, Varma RP, Sylaja PN, Thomas B, Kesavadas C (2017). Post-stroke cognitive impairment - A cross-sectional comparison study between mild cognitive impairment of vascular and non-vascular etiology. J Neurol Sci.

[55] Prins ND, Scheltens P (2015). White matter hyperintensities, cognitive impairment and dementia an update. Nat Rev Neurol.

[56] Brickman AM, Muraskin J, Zimmerman ME (2009). Structural neuroimaging in Alzheimer’s disease do white matter hyperintensities matter?. Dialogues Clin Neurosci.

[57] Habes M, Erus G, Toledo JB, Zhang T, Bryan N, Launer LJ (2016). White matter hyperintensities and imaging patterns of brain ageing in the general population. Brain.

